# Understanding How and When Therapists Misstep: Navigating Engagement Challenges in Youth Mental Health Services

**DOI:** 10.1007/s10802-025-01297-y

**Published:** 2025-03-11

**Authors:** Hyun Seon Park, Kimberly D. Becker, Bruce F. Chorpita

**Affiliations:** 1 Department of Psychology, University of California Los Angeles, Franz Hall, Los Angeles, CA 90095 USA; 2 Department of Psychology, University of South Carolina, 1512 Pendleton Street, Columbia, SC 29208 USA

**Keywords:** Engagement, Professional-patient relations, Psychotherapy, Supervision, Psychotherapeutic process, Community mental health services

## Abstract

Therapist “missteps” (i.e., inadvertent, less optimal, or noncollaborative therapist behaviors) have the potential to negatively impact youth and family engagement in community mental health services. The present study explores potential misstep occurrences and whether they varied across various distal and proximal factors related to treatment planning, preparation, and clinical context. Data were drawn from a multi-site cluster-randomized controlled trial focused on promoting therapist use of evidence-informed procedures to engage youth and families in urban and rural community mental health services. Audio recordings from 391 treatment sessions delivered by 92 therapists were transcribed and coded for missteps occurrences. Missteps were sporadic, but occurred in most sessions, frequently manifesting as advice giving, but taking a variety of other forms. Their occurrence appeared to be lower when supervision involved preparing a plan for engaging the client, when there was a single participant in the session, and when treatment sessions occurred soon after supervision. Engaging in preparatory activities in supervision and receiving timely supervision prior to treatment delivery may be more protective against therapist missteps than simply selecting a practice to deliver. Additionally, unique challenges of family sessions may increase the likelihood of missteps occurring, highlighting the importance of specialized training for conducting family sessions.

Low treatment engagement remains a pervasive issue within youth and family mental health services. Engagement challenges pose notable barriers to therapist clinical decision-making and have the potential to interrupt the delivery of evidence-based care. An overwhelming majority (96.7%) of mental health professionals in community mental health settings report encountering treatment engagement challenges in their caseload, including missed appointments, low homework completion, difficulty building or sustaining a positive relationship, and skepticism about the effectiveness of therapy (Becker et al., 2021a, b). Engagement challenges may be manifested in client behaviors that have been shown to tax therapists’ attention during a treatment event, such as minimal verbal and nonverbal responses or complaints about the therapist or in-session activities (Colli et al., 2019; Eubanks et al., 2015). Provision of negative feedback from clients, particularly when expressed without a clear strategy on how to adjust the treatment approach, has been shown to elicit feelings of guilt, anxiety, incompetence, and irritation from the therapist (Coutinho et al., 2011; Hill et al., 1996; Hill, 2020; Moltu et al., 2010) and subsequently may make it difficult for therapists to respond effectively in the moment (Kluger & DeNisi, 1996). However, engagement challenges may not be transparent to therapists (Becker et al., 2021a, b; Chorpita et al., 2024), thereby contributing to a clinical context in which therapists are unaware of the client’s low engagement and might even inadvertently behave in ways that strain engagement further. In one study, even when therapists are aware of engagement challenges, nearly two-thirds were unable to identify a well-suited solution to address the presenting engagement concern (Becker et al., 2021a, b).

Despite the ubiquitous nature of these engagement challenges and their documented impact on treatment outcomes and additional costs to mental health professionals and systems (Becker & Chorpita, 2023; Danko et al., 2016; Pellerin et al., 2010), practical guidance on how to navigate these challenges remains limited. Effective management of treatment engagement challenges in youth and adolescent mental health services requires a coordinated effort to accurately identify and implement a treatment plan targeting the presenting engagement challenges. However, there are major challenges when identifying which engagement challenges are present and subsequently selecting and preparing to deliver an evidence-based practice to target those specific engagement challenges.

First, the detection of engagement challenges is limited, given the multifaceted and dynamic nature of treatment engagement. Although almost entirely measured as a unidimensional construct (e.g., attendance or homework), treatment engagement has been shown to follow an empirically validated five-factor structure (Chorpita & Becker, 2022) involving dimensions of Relationship, Expectancy, Attendance, Clarity, and Homework (REACH; Becker et al., 2018). Additionally, treatment engagement challenges are *dynamic* (e.g., fluctuating between and within treatment events) and *transactional* (e.g., co-constructed by the therapist, youth, and family throughout treatment) (Becker et al., 2018; Chorpita & Becker, 2022; Haine-Schlagel & Walsh, 2015; Pullmann et al., 2013; Staudt et al., 2012), making them harder to detect at any given time. Not surprisingly, this complexity presents particular challenges regarding best practices, given that these five emergent and transactional dimensions have been shown empirically to respond to different types of procedures (Becker et al., 2021a, b). Moreover, observational coding of therapist use of engagement practices found therapists typically use only a limited set of engagement-focused practices (e.g., psychoeducation, rapport building, therapist reinforcement) which effectively target some domains of engagement (i.e., *Relationship*, *Attendance, Clarity*), but do not address others (i.e., *Expectancy, Homework*) (Wu et al., 2022). Consequently, navigating engagement challenges in service contexts often resembles traveling along a windy road, full of blind curves and missing “guardrails” to guide therapists, and can contribute to the occurrence of potential “missteps,” or less optimal and potentially “risky” therapist behaviors over the course of treatment delivery.

## Therapist “Missteps”

The class of therapist behaviors that are unintentional, less optimal, or noncollaborative, which we will henceforth refer to as “missteps,” are well-documented, as is their impact on client engagement, but are represented by a variety of terms. Colli and colleagues (2009; 2019) described therapist use of “negative interventions” (e.g., therapist hostility, use of technical jargon, criticism, unwanted advice-giving), which were associated with greater client indicators of ruptures in the therapeutic alliance (e.g., strong disagreement with therapist). Another study by Eubanks and colleagues (2019) demonstrated that ratings of “therapist contribution” to within-session alliance ruptures (i.e., the extent to which the therapist caused or exacerbated the rupture) predicted client dropout from services. Qualitative interviews conducted by Piselli and colleagues (2011) also shed light on therapist self-reported behaviors leading to premature termination, including “mistakes,” (e.g., failing to recognize or address a problem in treatment, offering too much advice, allowing personal feelings about a client to interfere with treatment), “disagreements about treatment” (e.g., conflicts that negatively impacted the therapeutic relationship), and “therapist emotions that presented a challenge” (e.g., feelings of frustration, discouragement, or burnout) (Piselli et al., 2011). Additional research has examined the misapplication of otherwise effective therapist strategies and techniques such as humor (Sarink & García-Montes, 2023; Valentine & Gabbard, 2014), inappropriate self-disclosure (Bottrill et al., 2010; Hanson, 2005), and advice-giving (Duan et al., 2018; Hill et al., 2023); and therapist behaviors characterized as unresponsive (Elkin et al., 2014), confrontational (Ackerman & Hilsenroth, 2001) or hostile (Colli et al., 2019). In addition to ruptures in the therapeutic relationship and premature termination, other adverse impacts of missteps are widespread, including interference with client progress (Henry et al., 1990); feeling unheard and unhelped by the therapist (Knox et al., 2011), decreased openness (Park et al., 2024); and abrupt or problematic termination from services (Renk & Dinger, 2002; Knox et al., 2011; Park et al., 2024).

Therapist missteps have been observed in diverse clinical contexts, including in the treatment of adult clients of racial, ethnic, sexual, or gender minority identities (Durham, 2018; Hook et al., 2016; Mizock & Lundquist, 2016; Owen et al., 2019; Spengler et al., 2016; Sue et al., 2007) or particular clinical presentations, such as personality disorders (Greaves, 1988) and eating disorders (Thompson & Sherman, 1989). Therapist missteps were also found to occur across delivery of specific treatment modalities, including Acceptance and Commitment Therapy (Brock et al., 2015) and psychodynamic therapy (Trimboli et al., 2016) and among mental health professionals of different training backgrounds spanning trainees, study therapists, and community providers (Knox et al., 2022; Kottler & Carlson, 2013; Buckley et al., 1979).

Although existing research in this area has focused predominantly on treatment with adult populations, a growing line of research has sought to measure potential therapist missteps when delivering care to youth and families (e.g., O’Keeffe et al., 2020; Park et al., 2024) and identify the context in which these missteps occur. In one study, adolescents who prematurely terminated from services identified similar therapist missteps to those documented with adult populations, such as minimal responses from the therapist, rigid adherence to an activity or session agenda, and focus on risk over other clinical concerns (O’Keeffe et al., 2020). In another study, clinical supervisors in youth community mental health settings posited that therapist missteps might vary according to features of the clinical context such as session participant type (e.g., youth-only, caregiver-only, or family) and therapist preparedness to respond in the moment to engagement challenges (Park et al., 2024). However, a historical focus on therapist attributes (i.e., which therapists are prone to errors), as opposed to contextual or situation attributes (i.e., what makes anyone prone to errors), has left many critical questions unanswered regarding these potential clinical missteps (see Budge, 2016).

One proposed hypothesis for why missteps may occur frequently in the context of low youth and family treatment engagement has posited the role of inadequate guidance for therapists to effectively identify and navigate engagement challenges as they unfold within a treatment session. Recent studies found that in community mental health contexts, therapists working with clients presenting with treatment engagement challenges were significantly less likely to receive guidance during supervision on identifying, selecting, and preparing a targeted plan for an engagement problem compared with therapists who received engagement-related clinical decision-making supports (Chorpita et al., 2024). Inadequate guidance in planning (e.g., identifying and selecting engagement practices) and preparing (e.g., role-playing, modeling, reviewing written materials) to target specific engagement challenges in clinical supervision may limit therapists’ ability to manage additional complexities and demands that may emerge within a treatment event (e.g., youth refusal to participate in a planned session activity, in-session conflict between youth and caregiver). For example, clients experiencing engagement challenges (e.g., ruptures in the therapeutic relationship, low expectancy about effectiveness of treatment) have been observed to exhibit a range of in-session responses to therapist behaviors or interventions. As such, identifying when therapist missteps may more frequently occur is a crucial next step to improving the quality of care for youth and families in community settings and illuminating remaining “blind curves” in need of decision-making supports to manage missteps when they occur.

## Present Study

The aim of this study was to investigate *how* and *when* therapists may misstep in therapy with youth and caregivers who have reported treatment engagement concerns. Regarding “how,” we first examined the types of missteps that occur in therapy and their relative frequencies. Then, we examined “when” missteps occur by considering proximal and distal factors that might be associated with missteps. Proximal factors included session participant type (i.e., youth-only, caregiver-only, family), misstep recipient (e.g., youth, caregiver), and therapist use of evidence-based structured clinical procedures immediately prior to the misstep. We expected that given additional clinical complexity, family sessions would be associated with greater misstep occurrences compared with youth-only or caregiver-only sessions. Additionally, it was hypothesized that misstep occurrences would frequently occur following less structured moments in the treatment event (i.e., in the absence of any identifiable evidence-based structured clinical procedures).

We examined two distal factors which represented activities that occurred in supervision prior to the treatment event itself: (1) selecting a specific practice to use in the next treatment session and (2) reviewing or rehearsing the selected practice. We also examined the days elapsed between supervision and treatment to examine how supervision recency to treatment might be associated with missteps. We expected that there would be fewer missteps in treatment events when therapists had selected and reviewed/rehearsed a specific practice in the preceding supervision. We also hypothesized that days elapsed between supervision and treatment would be positively associated with misstep frequency in treatment.

## Method

### Study Context

The data source included digital recordings of therapy sessions obtained from a multisite randomized controlled trial (RCT) testing an intervention designed to improve the use of case-based and research evidence in clinical decisions and actions related to youth and family treatment engagement (Chorpita et al., 2024). The RCT took place in the context of school mental health services delivered in publicly funded youth community mental health organizations located in two geographically distinct, under-resourced communities where service inequities are common. Therapists in the experimental condition were provided with a coordinated knowledge system (see Chorpita & Daleiden, 2018) and a 2-day training including use of measures to detect and prioritize multidimensional engagement concerns, review of written guides for 11 discrete clinical procedures with empirical support for engagement, and rehearsal of a one-page worksheet to support planning and monitoring in supervision and application of engagement-focused procedures in treatment events. Therapists in the enhanced care condition were alerted of each case in which either the youth or caregiver had reported an engagement concern and were provided with a set of written guides detailing the same 11 practices provided to the experimental condition resembling standard practice guides (i.e., limited to definitions and without thorough step-by-step instructions) and 1-day training event. Study procedures included audio-recording multiple face-to-face supervision events in which a supervisor-therapist dyad discussed a youth case who was potentially at risk for low treatment engagement based on youth and/or caregiver report on a treatment engagement survey. Study procedures also included audio-recording multiple treatment events in which there was therapeutic contact between the therapist and a youth and/or caregiver (most often face-to-face but possibly over the phone). Flow of digitally recorded supervision and treatment events could be depicted as S1 – T1 – S2 – T2 – S3. Supervision events were observationally coded for the presence of therapist activities related to practice selection and preparation using the Action Cycle and Use of Evidence Behavioral Observational Coding System (ACEBOCS, Park et al., 2020). All study procedures were reviewed and approved by the Institutional Review Boards (IRB) of University of California, Los Angeles and the University of South Carolina, as well as the IRBs of the participating service organizations that requested independent reviews. The study design, sample and recruitment, and primary outcomes have been elaborated in detail in previous publications (Chorpita et al., 2024); thus, this paper includes details that are specifically relevant to its research questions about missteps.

The present study’s focus on the type and frequency of therapist missteps and proximal factors (i.e., session participant type, misstep recipient) relied largely on the coding (described below) of transcribed audio recordings of treatment events as the data source. Therapist use of structured clinical procedures, the remaining proximal factor, was also derived from treatment events, and its coding has been described elsewhere (see Park et al., 2024). Data on distal factors (i.e., practice selection and review/rehearsal) were derived from transcribed and coded supervision events, the coding of which has been described in other publications (see Chorpita et al., 2024). Thus, we will elaborate on coding specific to missteps in this paper and share only brief details related to coding and findings that have been published elsewhere.

### Treatment Event Recording Sample

For each youth case enrolled in the RCT, the therapist audio-recorded one to four treatment events (*M* = 1.88, *SD* = 0.46), yielding a total of 392 recordings that were transcribed and qualitatively coded for therapist missteps. One recording was excluded as no caregiver or client was present (i.e., the therapist recorded a voicemail in which they provided an appointment reminder to the caregiver), yielding 391 audio-recorded treatment events and corresponding transcripts in the present sample.

### Participants

#### Therapists

Table 1 displays the background characteristics, collected on a survey administered at the start of the RCT, of the 92 therapists with audio-recorded treatment events in the present study. In general, therapists were women in their middle years from ethnic-racial minoritized groups. Therapists reported practicing primarily from a cognitive-behavioral orientation (64.1%), and mostly commonly reported receiving formal training in Trauma-Focused Cognitive Behavioral Therapy (58.7%), Managing and Adapting Practice (42.4%), and Seeking Safety (38.0%).

**Table 1:** Participant characteristics

Variable	Therapists	Clients
*N*	92	209
Age in years,* M (SD)*	38.4 (9.9)	12.7 (3.5)
Gender		
Female	84 (91.3%)	95 (45.5%)
Male	8 (8.7%)	114 (54.5%)
Race/Ethnicity		
Latinx	42 (45.7%)	94 (45.0%)
Black	36 (39.1%)	72 (34.4%)
White	12 (13.0%)	40 (19.1%)
Asian	3 (3.3%)	2 (1.0%)
Unknown		1 (0.5%)
Years of Since Degree,* M (SD)*	6.4 (5.6)	
Years of Workplace Experience, *M (SD)*	4.5 (4.8)	
Caseload,* M (SD)*	26.9 (17.8)	
Highest Degree Obtained		
Master of Social Work	56 (60.9%)	
Master of Arts / Sciences	33 (35.9%)	
Bachelor of Arts / Sciences	2 (2.2%)	
Doctoral Degree	1 (1.01%)	
Primary Theoretical Orientation		
Cognitive Behavioral	59 (64.1%)	
Eclectic	18 (19.6%)	
Family Systems	5 (5.4%)	
Humanistic/Client-Centered	5 (6.5%)	
Psychodynamic	1 (1.1%)	
Other	3 (3.3%)	
Formal Training in Evidence-Based Practices		
Trauma Focused Cognitive Behavioral Therapy	54 (58.7%)	
Managing and Adapting Practice	39 (42.4%)	
Seeking Safety	35 (38.0%)	
Cognitive Behavioral Intervention for Trauma in Schools	20 (21.8%)	
Positive Parenting Program	12 (13.0%)	
Parent Child Interaction Therapy	6 (6.5%)	
Child Parent Psychotherapy	6 (6.5%)	
Incredible Years	3 (3.3%)	
Interpersonal Psychotherapy	3 (3.3%)	
Primary Problem		
Mood Related		53 (25.4%)
Attention Related		43 (20.6%)
Disruptive Behavior Related		39 (18.7%)
Anxiety Related		31 (14.8%)
Trauma Related		25 (12.0%)
Other Related		17 (8.1%)
Unknown		1 (0.5%)

#### Youth and Caregivers

Table 1 also displays the characteristics of the 209 youths whose audio-recorded treatment events were part of the present study. As noted in electronic health records, the youth sample was mostly in their adolescent years. Slightly less than half the youths were female and most were either Latinx or Black. Clinical concerns, also obtained from electronic health records, indicated that the sample sought help for problems that are typical in school mental health settings (Rones & Hoagwood, 2000). Because our study team had no direct contact with youth and families, we did not obtain background information on caregivers (e.g., relationship, age, marital status).

### Misstep Coding

A total of 391 treatment transcripts were transcribed and qualitatively coded using a two-phase coding system to detect missteps. Qualitative research design and a codebook thematic analysis approach were utilized to analyze data derived from transcripts of individual therapy sessions (Braun & Clarke, 2022).

#### Phase I Coding

In the first phase, coders for the larger study trial conducted a review of treatment transcripts to detect behaviors that might be considered missteps using the Use of Research Evidence Content Application-Engagement (URECA-E), a behavioral observation coding system designed to measure therapists’ behaviors that supported client treatment engagement. Based on a modified version of the PracticeWise Clinical Coding System (PracticeWise, 2023), URECA-E has been utilized in prior reviews of engagement interventions and studies (i.e., Becker et al., 2015a, b; Becker et al., 2015a, b; Becker et al., 2018; Lindsey et al., 2014). This coding system included a general “misstep” code that captured behaviors potentially unsupportive of treatment engagement, operationalized as “intervention-agnostic therapist behaviors that (1) occurred in the presence of engagement challenges and (2) are considered to be potentially risky to youth and caregiver engagement.” This code demonstrated good interrater reliability (κ = 0.62). Across the 391 treatment events, 224 (57.3%) contained at least one excerpt coded with a possible misstep, and a total of 894 excerpts were coded as a possible misstep. Then, after all treatment transcripts had been coded in the first phase, the second phase of coding involved a more granular specification of the nature of each misstep that had been coded into the general misstep category.

#### Phase II Coding

##### Codebook

This phase of coding involved an elaborated codebook of potential missteps. Codebook development involved generating initial codes and definitions deductively based on literature reviews on misstep-related constructs, including ruptures (Eubanks et al., 2018), negative therapeutic processes (e.g., shame; Black et al., 2013), and misapplication of therapeutic techniques (e.g., misuse of humor; Sarink & García-Montes, 2023; Valentine & Gabbard, 2014). Then, an inductive, data-driven approach involving a review of a subset of treatment events identified as containing at least one misstep occurrence (*n* = 162, 72%) was utilized to refine the codebook if the content of a misstep occurrence did not fit the deductively developed codes and definitions. Member checking with key informants (McKim, 2023) with extensive years of experience at their respective study sites (*M* = 16.67*, SD* = 4.73*)* was conducted through structured interviews to take a non-colonial approach to understand and validate potential misstep codes (Park et al., 2024).

The final codebook consisted of 10 unique misstep codes and definitions: (1) *advice-giving* (e.g., direct persuasion, telling the recipient what to do without teaching, reinforcing, or consolidating a generalizable skill); (2) *arguing or displaying doubt/disbelief* (e.g., expressing diverging views typically in a heated or persistent manner); (3) *criticizing* (e.g., blaming or complaining about the recipient); (4) *demanding attention* (e.g., engaging in verbal bids for attention); (5) *downplaying problem, identity, beliefs* (e.g., minimizing or trivializing, putting a “positive spin” to a problem); (6) *eliciting shame/guilt* (e.g., using feelings of guilt or remorse to change how they think, feel, or act); (7) *highlighting negative consequences/appealing to fear to motivate change* (e.g., attempting to increase fear to motivate behavior change); (8) *inappropriate elicitation/disclosure of information* (e.g., disclosing information or anecdotes in a lengthy manner without typing back to client concerns); (9) *making a joke at family’s expense* (e.g., using inappropriate humor to make the recipient the target of the joke); (10) *speculating or making assumptions* (e.g., assuming or speculating without evidence, information, or checking in with the recipient). Full definitions are provided in Table 2.

**Table 2:** Misstep codes and definitions

Misstep Code	Definition
Advice-giving	Giving advice or using direct persuasion by telling the recipient what to do in a given situation, but without teaching, reinforcing, or consolidating generalizable skill
Arguing or displaying doubt/disbelief	Arguing or expressing diverging views, typically in a heated or persistent manner (e.g., persistent non-Socratic questioning, fact-checking)
Criticizing	Indicating perceived faults of recipient in a disapproving and judgmental way (e.g., preemptively blaming/accusing, negatively labeling words or behaviors, pointing out mistakes non-constructively, complaining about recipient)
Demanding attention	Engaging in verbal bids for attention from the recipient (e.g., interrupting, talking over, commanding, commenting on lack of engagement)
Downplaying problem, identity, beliefs	Minimizing or trivializing the recipient’s problem, identity, or beliefs (e.g., putting a “positive spin” on a problem, oversimplifying the problem and barriers to solutions)
Eliciting shame/guilt	Using feelings of guilt, remorse, or a sense of responsibility about recipients’ words or behaviors to change how they think, feel, or might act (e.g., implying recipient "owes" change to others, expressing disappointment, highlighting negative impact/burden of recipient's words or behaviors on others)
Highlighting negative consequences/ appealing to fear to motivate change	Highlighting negative consequences or attempting to increase fear to motivate behavior change in the recipient
Inappropriate elicitation/disclosure of information	Eliciting or disclosing information that is irrelevant to the recipient and their treatment (e.g., eliciting or disclosing personal information or anecdotes in a lengthy manner without tying back to client concerns, gossiping)
Making a joke at family's expense	Using humor directed at the recipient or recipient's situation using inappropriate humor (e.g., making recipient/situation the target of the joke, reacting inappropriately, commenting humorously)
Speculating or making assumptions	Assuming or speculating on recipient's presenting problems without evidence or information or checking in with the recipient

Adapted from Park et al., 2024

##### Coding Team and Procedures

The coding team consisted of five clinical psychology doctoral students of diverse racial, ethnic, and gender identities with clinical and research expertise in children’s mental health services and treatment engagement. All coders had clinical experience delivering care to youth of diverse racial, ethnic, and gender identities and their families in community contexts resembling both study sites. Coders were instructed to apply one code that best described the misstep present in the excerpt and were provided with the entire session transcript to provide contextual information about the session. During weekly coding meetings, any excerpt containing multiple types of missteps (i.e., *advice-giving* followed by *criticizing)* was excerpted further, and excerpts considered to not contain a potential misstep were excluded. Member checking with key informants was conducted through structured interviews to understand and validate the codes. Three mental health professionals, identified as local experts in the services sites of the larger study, were intentionally selected for this process and participated in a structured virtual interview with the lead doctoral student and two doctoral-level psychologists with expertise in children’s mental health services. During the interviews, key informants were presented with a sample of site-specific excerpts for each misstep code and then asked to describe the content of the excerpts, whether they believed these excerpts depicted a misstep, what reasons might have led to the behavior in the excerpt, and the impact of missteps on client care. Key informants were then asked to review and revise each misstep code and definition, which were incorporated into the final codebook.

Interrater reliability was calculated for 684 double-coded excerpts (69.9%), demonstrating good to very good reliability (Fleiss’ k = 0.71–0.88) across the 10 misstep codes according to guidelines outlined by Landis & Koch (1977). Additional information about the missteps codebook development and coding procedures can be found in Park et al. (2024).

#### Coding of Proximal and Distal Factors

##### Proximal Factors

Three proximal factors were examined in this study: session participant type, misstep recipient, and therapist use of evidence-based structured clinical procedures. Regarding *session participant type*, each treatment event was categorized based on who was present in the session with the therapist: youth-only (i.e., only youth present), caregiver-only (i.e., only caregiver), or family (i.e., youth and at least one caregiver were present). Misstep *recipient* (i.e., the individual the misstep was directed towards) was coded for each misstep occurrence and determined by examining the transcript text and speaker turns for each excerpt coded as a misstep. The coding of *use of evidence-based* structured clinical procedures has been described in detail elsewhere (Chorpita et al., 2024) and involved applying codes that represented discrete behaviors related to treatment engagement (e.g., providing verbal reinforcement, demonstrating a skill, eliciting a commitment). In the present study, we identified the code associated with the therapist behavior that preceded each therapist misstep and categorized it into one of three categories: (1) use of a structured clinical procedure to promote engagement (i.e., providing verbal reinforcement); (2) occurrence of another therapist misstep; and (3) absence of any discernable activities resembling structured clinical procedures or missteps (e.g., tangential conversations initiated by the therapist, youth, or caregiver).

##### Distal Factors

Three distal factors were examined in this study: practice selection, practice review/rehearsal, and days elapsed between supervision and the subsequent treatment event. As noted, *practice selection* and *review/rehearsal* were coded from supervision events and have been described in detail elsewhere (Park et al., 2020). In short, these two codes were utilized in the present study to indicate whether treatment planning (e.g., selecting a practice to deliver in the next treatment event) and preparation of an engagement practice (e.g., engaging in role-play, modeling, and reviewing of written materials appropriate to the practice) transpired in the most recent supervision event occurring before each treatment event. Thus, each supervision event was assigned two dichotomous codes (presence/absence): (1) the selection of an engagement practice (e.g., deciding to “address barriers to treatment” in the next treatment event) and (2) preparation to deliver an engagement practice (e.g., role-playing or modeling ways to “address barriers to treatment”). These codes were then associated with the subsequent treatment event for purposes of analysis. Additionally, the number of *days elapsed* between supervision and treatment was measured by the distance in days between the preceding supervision event and each treatment event.

### Data Analyses

Descriptive statistics on the frequency of misstep occurrences were obtained for general (i.e., *any misstep*) and specific misstep types (e.g., *advice-giving, criticizing*) to explore how rates of misstep occurrences may differ across the following proximal factors: (1) session participant type; (2) recipient and (3) specific therapist behaviors occurring before each misstep instance.

To investigate the association between total misstep occurrences and temporally distal and proximal factors, multilevel analyses with random intercepts were conducted using the “lme4” package in R (Bates, et al., 2015). Specific distal (i.e., practice selection, practice review/rehearsal, days elapsed between supervision and the subsequent treatment event) and proximal (i.e., session participant type) were included in the multilevel analyses. To more accurately reflect expected differences in misstep frequencies across study conditions, two granular, observationally coded variables that captured the key differences between the two conditions were included: (1) practice selection (i.e., whether the therapist selected an engagement practice in the following treatment event during supervision) and (2) practice review/rehearsal (i.e., whether the therapist engaged in activities to prepare to use that practice such as roleplaying or reviewing written materials). Using youth-only sessions as the reference group, two binary variables (i.e., caregiver session, family session) were created using dummy-coding techniques and added to the model to account for the levels in session participant type. Up to four levels of nesting were considered (i.e., treatment events nested within clients nested within therapists nested within supervisors). Null models were utilized to calculate the proportion of variance accounted for each level of nesting and examine model fit. Clients (level 2) and supervisors (level 4) were found to account for little variance in total misstep occurrences and did not significantly improve fit. The final model consisted of two levels: treatment events nested within therapists. Therapist caseload (i.e., number of active clients treated by each therapist) and number of evidence-based treatments (EBTs) for which the therapist received training were included as covariates to account for the variability in therapist familiarity and training in EBTs and active caseload size. Assumptions of multilevel modeling were met, including normality of residuals, homoscedasticity, and assumptions of linearity.

Hierarchical regression modeling was utilized to assess the unique contribution of each additional predictor to the variance in the total number of misstep occurrences. The hierarchical regression analysis proceeded in three steps. In the first step, the selection of an engagement practice, session participant type, and covariates were added. Preparation of an engagement practice was added in the second step, and days elapsed was added in the third and final step. Likelihood ratio tests were conducted, and it was determined that each iterative model accounted for significantly more variance in total misstep occurrences (*p* = 0.02; *p* = 0.04). By sequentially entering predictors into the regression model, the hierarchical approach facilitated the unique contribution of each predictor to the variance in total misstep occurrences, beyond the effects of previously entered variables.

## Results

Results are presented in order, with temporally distal risks presented first, followed by an in-depth exploration of temporally proximal factors related to in-session activities.

### Misstep Frequency Across Proximal Factors

Examining the 391 sessions (of which 224 had at least one misstep), we found that 5.8% of all therapist behaviors were coded as a misstep. On average, there were 2.50 (*SD* = 4.40) misstep occurrences in each session. Table 3 displays the relative frequencies of each misstep type (see Total column). *Advice-giving* was the most frequently occurring misstep type, accounting for 37.9% (*n* = 369) of all misstep occurrences, whereas *eliciting shame/guilt* occurred least frequently (3.5%; *n* = 34).

**Table 3:** Frequency and mean (SD) of missteps observed within sessions by participant type

		Session Participant Type					
Missteps Observed	Total (% of 979 occurrences)	Youth Only (231 events)		Caregiver Only (35 events)		Family (125 events)	
		*n*	*M (SD)*	*n*	*M (SD)*	*n*	*M (SD)*
Advice-giving	369 (37.9%)	189	0.82 (1.6)	31	0.89 (1.6)	149	1.19 (2.0)
Arguing or displaying doubt/disbelief	152 (15.5%)	75	0.32 (0.9)	7	0.20 (0.5)	70	0.56 (1.2)
Criticizing	109 (11.1%)	44	0.19 (0.5)	5	0.14 (0.5)	60	0.48 (1.7)
Demanding attention	44 (4.5%)	22	0.10 (0.4)	0	0	22	0.18 (0.8)
Downplaying problem, identity, beliefs	56 (5.7%)	42	0.18 (0.7)	1	0.3 (0.2)	13	0.10 (0.4)
Eliciting shame/guilt	34 (3.5%)	14	0.06 (0.4)	0	0	20	0.16 (0.8)
Highlighting negative consequences/ appealing to fear to motivate change	71 (7.3%)	40	0.17 (0.5)	2	0.06 (0.2)	29	0.23 (0.8)
Inappropriate elicitation/disclosure of information	52 (5.3%)	22	0.10 (0.7)	2	0.06 (0.2)	28	0.22 (0.6)
Making a joke at family's expense	36 (3.7%)	10	0.04 (0.2)	0	0	26	0.21 (0.5)
Speculating or making assumptions	56 (5.7%)	44	0.19 (0.6)	1	0.03 (0.2)	11	0.09 (0.4)
Any Misstep	979	502	2.17 (3.6)	49	1.4 (2.4)	428	3.42 (5.9)

One treatment event was excluded given the nature of the recording (i.e., recording of therapist’s voicemail to caregiver, with no youth or caregiver present)

#### Session Participant Type

Across the 391 sessions, 231 (59%) sessions were youth-only, 35 (9.0%) were caregiver-only sessions, and 125 (32.0%) sessions were family sessions (i.e., at least one caregiver and youth present). Across these session participant types, 127 (55.0%) youth-only sessions, 16 (45.7%) caregiver-only sessions, and 81 (64.8%) family sessions contained at least one misstep. Descriptive statistics on the frequency of misstep occurrences organized by misstep and session participant types are provided on Table 3. Family sessions on average had the highest number of misstep occurrences per session (*M* = 3.42, *SD* = 5.9), followed by youth-only sessions (*M* = 2.17, *SD* = 3.6) and caregiver-only sessions (*M* = 1.4, *SD* = 2.4). Within each session participant type, misstep occurrences also varied by misstep type (see Youth-Only, Caregiver-Only, and Family columns of Table 3). Specifically, across the 231 youth-only sessions, 502 total misstep occurrences spanning all 10 misstep types were coded, with *advice-giving* as the most frequent misstep type (37.6%; *n* = 189) and *making a joke at the family’s expense* occurred least frequently (2.0%; *n* = 10). In contrast, across the 35 caregiver-only sessions, seven misstep types accounted for the 49 misstep occurrences coded, and just five misstep types were found to have occurred more than once. The most frequent misstep type in caregiver-only sessions was *advice-giving* (63.3%; *n* = 49). Across the 125 family sessions, 428 total misstep occurrences spanning all 10 misstep types were coded. *Advice-giving* was the most frequent misstep type (34.8%; *n* = 149) and *speculating or making assumptions* occurred least frequently (2.6%; *n* = 11).

#### Recipient

Frequencies of misstep occurrences in family sessions organized by the recipient of the misstep (i.e., youth-, caregiver-, or family-directed) are displayed in Table 4. Across the 428 misstep occurrences in family sessions, 66% (*n* = 284) of misstep occurrences were directed at the youth, 29% (*n* = 124), were directed toward the caregiver, and 5% (*n* = 20) were directed at the family (i.e., both the youth and caregiver). Eight of the 10 misstep types were directed most frequently at the youth only, and the remaining two misstep types (i.e., *inappropriate elicitation/disclosure of information*, *speculating or making assumptions*) were directed more frequently at the caregiver only.

**Table 4:** Frequency of missteps observed in family sessions by recipient

	Family Sessions (125 events)		
Missteps Observed	Youth-Directed	Caregiver-Directed	Family-Directed
Advice-giving	84 (56%)	62 (42%)	3 (2%)
Arguing or displaying doubt/disbelief	53 (76%)	16 (23%)	1 (1%)
Criticizing	46 (77%)	10 (17%)	4 (7%)
Demanding attention	18 (82%)	4 (18%)	0
Downplaying problem, identity, beliefs	10 (77%)	2 (15%)	1 (8%)
Eliciting shame/guilt	18 (90%)	2 (10%)	0
Highlighting negative consequences/ appealing to fear to motivate change	25 (86%)	4 (14%)	0
Inappropriate elicitation/disclosure of information	10 (36%)	16 (57%)	2 (7%)
Making a joke at family's expense	17 (65%)	3 (12%)	6 (23%)
Speculating or making assumptions	3 (27%)	5 (45%)	3 (27%)
Any Misstep	284 (66%)	124 (29%)	20 (5%)

Denominator for percentages are total occurrences of each misstep type in family sessions

#### Therapist Behaviors Preceding Misstep Occurrences

Frequencies of therapist behaviors preceding misstep occurrences are outlined in Table 5. It is important to note that these frequencies do not demonstrate associations between misstep occurrences and the preceding therapist behaviors. Broadly, 60.2% (*n* = 632) of misstep occurrences followed the therapist's use of structured clinical procedures, such as *express empathy*, *engage in noncontingent positives*, or *elicit perspectives about the problem* during the session. One-quarter of missteps (*n* = 253; 25.2%) occurred following instances characterized by an absence of both a structured clinical procedure or misstep occurrences (i.e., *no codes applicable*). Consecutive misstep occurrences (e.g., *criticizing* followed by *advice-giving*) accounted for 10.6% of total misstep occurrences (*n* = 105). Overall, *advice-giving* following the use of a structured clinical procedure was the most frequently occurring scenario and accounted for 25.9% (*n* = 254) of all misstep occurrences. *Arguing or displaying doubt/disbelief* following the use of a structured clinical procedure was the next most frequent (10.5%, *n* = 103), followed by *advice-giving* in the absence of any discernable activities (8.4%, *n* = 82). Nine of the 10 misstep types occurred most frequently following therapist’s use of an evidence-based strategy while *making a joke at the family’s expense* occurred most frequently in the absence of evidence-use or misstep occurrences.

**Table 5:** Frequency of missteps observed by preceding therapist behavior type

	Preceding Therapist Behavior		
Missteps Observed	Clinical Procedure (% of 632 instances)	Any Misstep (% of 105 instances)	No Codes Applicable (% of 253 instances)
Advice-giving	254 (40.6%)	33 (31.7%)	82 (32.9%)
Arguing or displaying doubt/disbelief	103 (16.5%)	12 (11.5%)	37 (14.9%)
Criticizing	66 (10.5%)	13 (12.5%)	30 (12.0%)
Demanding attention	32 (5.1%)	3 (2.9%)	9 (3.6%)
Downplaying problem, identity, beliefs	34 (5.4%)	7 (6.7%)	15 (6.0%)
Eliciting shame/guilt	16 (2.6%)	5 (4.8%)	13 (5.2%)
Highlighting negative consequences/ appealing to fear to motivate change	38 (6.1%)	16 (15.4%)	17 (6.8%)
Inappropriate elicitation/disclosure of information	27 (4.3%)	5 (4.8%)	20 (8.0%)
Making a joke at family's expense	26 (4.2%)	2 (1.9%)	28 (3.2%)
Speculating or making assumptions	30 (4.8%)	8 (7.7%)	18 (7.2%)
Any Misstep (% of 979 instances)	632 (63.2%)	105 (10.6%)	253 (25.2%)

Two instances in which the misstep was not preceded by any therapist behavior (i.e., treatment event began with a therapist missteps) were captured under *No Codes Applicable*

Six clinical procedures were found to frequently occur before misstep occurrences: (1) *elicit perspective about the problem*, (2) *engage in noncontingent positives*, (3) *express empathy*, (4) *make a specific plan,* (5) *reward: social*, and (6) *elicit perspectives about treatment*. Notably, therapist elicitation of the youth or family’s perspectives on the presenting problems (*elicit perspectives about the problem*) was found to occur immediately before instances of all 10 misstep types and accounted for 17.4% (*n* = 170) of all misstep occurrences and 10.3% of all occurrences of this specific clinical procedure across the entire sample. Additionally, therapist efforts to create a specific plan of action for the therapist, youth, or caregiver (*make a specific plan*) were observed to occur before misstep types characterized by confrontation and elicitations of change (i.e., *arguing or displaying doubt/disbelief, criticizing, eliciting shame/guilt, highlighting negative consequences or appealing to fear to motivate change*) and accounted for 3.2% (*n* = 31) of all misstep occurrences and 3.4% of all occurrences of this clinical procedure.

### Distal and Proximal Predictors of Total Misstep Occurrences

Results are listed in Table 6. In the first model tested, only session participant type (i.e., family sessions compared with youth-only-sessions) was found to be a significant predictor of total misstep occurrences (*p* = 0.01). The intercept was also significant, suggesting that the expected occurrence of missteps in a youth-only session for which the therapist did not make a plan to use an engagement practice is approximately 2.3 missteps (*p* < 0.001). In the second model tested, session participant type (i.e., family sessions compared with youth-only sessions) and therapist preparation to deliver an engagement practice were found to significantly predict total misstep occurrences (*p* = 0.01; *p* = 0.02, respectively). The intercept was also significant, suggesting that youth-only sessions delivered by therapists who did not make a specific plan and did not prepare to deliver an engagement practice were expected to contain approximately 2.7 missteps (*p* < 0.001).

**Table 6:** Hierarchical regression analysis of predictors of total misstep occurrences

Predictor	*Model 1*	*Model 2*	*Model 3*
(Intercept)	2.32***	2.65***	2.29***
Caregiver Session	0.19	0.10	0.04
Family Session	1.26**	1.23**	1.19*
Therapist Caseload	0.01	0.01	0.01
Number of Trained EBTs	−0.33	−1.30	−0.26
Engagement Practice Planned	−0.52	−0.03	−0.05
Engagement Practice Prepared		−1.24*	−1.24*
Days Elapsed Between Supervision and Treatment			0.04*
*R*^*2*^* (Session-Level)*	0.04	0.06	0.07
*R*^*2*^* (Therapist-Level)*	0.26	0.25	0.25
Model Comparison	*∆R*^*2*^	LRT (df)	*p*
Regression 1 vs. Regression 2	0.02	5.65 (1)	.02*
Regression 2 vs. Regression 3	0.01	4.26 (1)	.04*

*** *p* < .001, ** *p* < .01, * *p* < .05**.** LRT = Likelihood Ratio Test. The LRT statistic compares the fit of nested models. Significant p-values indicate that the addition of predictors significantly improves model fit

In the third and final model tested, session participant type (i.e., family sessions compared with youth-only sessions), therapist preparation to deliver an engagement practice, and days elapsed between supervision and treatment were all found to be significant predictors of total misstep occurrences. Selecting an engagement practice to deliver at the next treatment event was not found to significantly predict total misstep occurrences; however, therapists who prepared to deliver an engagement practice were found to engage in approximately 1.2 fewer missteps than therapists who did not prepare to deliver an engagement practice during supervision (*p* = 0.02). Days elapsed between supervision and treatment was also found to significantly associated with total misstep occurrences, with each unit increase (i.e., days elapsed from supervision to treatment) associated with an incremental increase in misstep occurrences (*p* = 0.04). Family sessions were found to contain significantly more misstep occurrences compared with youth-only sessions, with family sessions containing approximately 1.2 more missteps than youth-only sessions, controlling for the selection and preparation of an engagement practice and days elapsed (*p* = 0.01). Lastly, the intercept was significant (*p* < 0.001), suggesting that in youth-only sessions occurring on the same day as a supervision meeting during which no engagement practices were selected or prepared, delivered by a therapist trained on an average number of EBTs and an average caseload, the expected occurrence of missteps is approximately 2.3 missteps per session.

## Discussion

The present study sought to investigate *how* and *when* therapists may potentially misstep in therapy with youth and caregivers experiencing treatment engagement challenges. In this initial effort, we sought to examine potential therapist missteps as a construct relevant to therapist effectiveness and treatment engagement in youth community mental health settings. However, given the relative novelty of this construct, particularly in youth mental health settings, it is important to acknowledge its evolving nature and the potential for variability in how they may present across clinical settings (e.g., therapeutic modalities, populations, settings) and their potential impact on relevant outcomes. In the present study, a broad, intervention-agnostic definition of therapist missteps was selected to allow for broader application across treatment settings and evidence-based practices, in hopes of offering preliminary insights to support additional research to refine this construct and expand its application to diverse clinical contexts.

Regarding *how* therapists may misstep, the occurrence of misstep types and their relative frequencies across various proximal factors were examined. Findings suggest that while missteps were a common occurrence across sessions, with over half of the recorded sessions containing at least one misstep occurrence, missteps represented a small portion (5.8%) of all recorded therapist behaviors within these sessions, suggesting that much remains unclear about when these missteps may happen within a session. Additionally, misstep occurrences were observed to occur in more than half of family and youth-only sessions, and just under half of caregiver-only sessions, with an average of approximately 2.5 missteps per session across session participant type. These findings are in line with the existing literature suggesting that less optimal and potentially noncollaborative therapist behaviors are a common occurrence in various treatment contexts and present initial efforts to expand the reach of this growing body of research (which has focused predominantly on adult populations) by examining how missteps may present and unfold in youth and family treatment contexts. Additionally, the high rate of potential missteps observed across treatment events could pose many potentially iatrogenic treatment outcomes, including premature termination prior to achieving expected treatment gains, exacerbating strain in youth and caregivers who are already reporting treatment engagement concerns, and decreasing future help-seeking behaviors.

Closer examination of recipients of missteps in family sessions highlights that most misstep occurrences (66%) are directed towards the youth only. This pattern held across all misstep types, apart from two misstep types (i.e., *Speculating or making assumptions* and *Inappropriate elicitation/disclosure of information*), which in family sessions were more frequently directed at the caregiver only. Notably, missteps were rarely directed at the family (i.e., youth and the caregiver simultaneously), suggesting that missteps are more likely to be directed at only one individual at a time and may arise from challenges balancing therapist alignment with the youth and the caregiver when both are present during family sessions (Park et al., 2024).

Over one-third of missteps occurred following another misstep (i.e., consecutive misstep occurrences) or instances where no structured clinical procedure or misstep was observed (i.e., *no codes applicable*). In addition, several clinical procedures, characterized by open-ended discussions, activities, or reinforcing statements to increase treatment engagement were also found to occur frequently before misstep occurrences. These findings align with prior findings highlight the additional decision-making demands when intervening on engagement challenges and suggest that therapists may be more prone to misstep in less structured moments of a session.

Regarding *when* missteps occurred, the present study identified several significant distal and proximal predictors of misstep occurrences in youth and family mental health services, shedding light on factors that may confer a greater risk of misstep occurrences when delivering treatment to youth and families presenting with engagement challenges. First, session participant type emerged as a significant predictor of misstep occurrences, underscoring the unique challenges and dynamics present specifically in family sessions (where multiple individuals are participating in treatment), which may increase the likelihood of missteps occurring. This is consistent with our findings that family sessions had the highest average of misstep occurrences (approximately 3.4 missteps per session), and with interviews with our community partners, who posited that the additional complexity posed by family sessions due to multiple alliances to maintain and lack of adequate training on navigating family sessions could leave therapists more vulnerable to engaging in inadvertent missteps (Park et al., 2024). This is further reflected in the training history of therapists in the present study, in which only 5.4% of therapists reported a primary family systems theoretical orientation and overall reported receiving the least training in evidence-based treatments that involve family members in treatment (e.g., PCIT, CPP, Incredible Years). The involvement of multiple individuals with varying needs, engagement challenges, and treatment perspectives may contribute to this observed difference in misstep occurrences in family sessions compared with youth-only sessions and is consistent with the documented complexities of conducting family therapy (Pope & Tabachnick, 1993; Rober, 2011; Wilson, 2007).

Furthermore, our analysis revealed that *selecting* an engagement practice during supervision to deliver in the following treatment event did not significantly predict total misstep occurrences on its own. However, *preparing* to deliver an engagement practice that was selected in the preceding supervision was significantly associated with a reduction in misstep occurrences during sessions. These findings may suggest that actively engaging in preparatory activities to deliver an engagement practice may be more protective against misstep occurrences than simply selecting which practice to deliver. Therapists who prepared to implement engagement practices with their supervisor before the treatment event by engaging in activities such as reviewing the steps involved in the engagement practice or role-playing demonstrated a lower frequency of misstep occurrences per session compared with therapists who did not engage in these preparatory activities. This finding aligns with prior research indicating these supervision activities significantly impact therapist behaviors and the use of evidence-based clinical procedures in subsequent treatment sessions (e.g., Bearman et al., 2013; Dorsey et al., 2016).

In the absence of such preparation activities, therapists may find themselves needing to make “run time” decisions (see Chorpita & Daleiden, 2018), where they must respond flexibly and immediately to presenting engagement concerns as they unfold within a session (e.g., by deviating from the established treatment plan or intervening on a new engagement concern). Such run-time decisions, in which therapists must make a *specific* clinical decision in a *particular* moment with a *particular* client and their presenting concerns, are inherently limited by the therapist’s existing knowledge available to them at that moment (e.g., training background, the selected treatment manual, or protocol). Consequently, such decisions may be more prone to inadvertent missteps as therapists do their best with limited guidance to predict what is the next best strategy (see Meehl, 1957), leaving them with vague boundaries separating appropriate from less appropriate variations of clinical practice (Dawes, 2005). Therefore, deliberate preparation of an engagement practice during supervision, particularly when treating youth and families with engagement challenges, appears to protect against inadvertent misstep occurrences, underscoring the important role of supervision in facilitating therapist preparedness and treatment delivery.

Additionally, the days elapsed between supervision and treatment was found to be significantly associated with misstep occurrences, indicating that longer duration between supervision and treatment events was linked to increased misstep occurrences. These results mirror previous studies, which have shown that fewer days elapsed between supervision and treatment (e.g., practicing a planned skill in supervision in a timely manner before the subsequent treatment event) has been shown to increase the transfer of training (Westman et al., 2020; Blume et al., 2010). As such, timely supervision may be crucial in maintaining therapeutic effectiveness and minimizing the occurrence of missteps.

It is important to note that therapist caseload and number of trained EBTs were not significant predictors of misstep occurrences, suggesting that reducing caseloads or increasing training in EBTs may not be the most effective solution to decrease misstep occurrences. Rather, allocating resources at the organizational level to support regular, timely supervision meetings and shaping supervision activities to include preparatory activities to support therapist knowledge, familiarity, and delivery of engagement practices may serve as effective “guardrails” for therapists, particularly when working with youth and families presenting with engagement challenges. Additional considerations may also include increasing training opportunities and therapist competencies in facilitating family sessions, which may be a particularly risky context for inadvertent misstep occurrences.

Although these results indicate a significant step towards identifying specific contexts in which therapist missteps occurred when treating youth and caregivers experiencing engagement challenges, a prevalent concern in youth mental health services, there are several limitations to consider. While frequencies of specific misstep types were coded for each treatment event, the analyses examined how distal and proximal factors may predict the total frequency of missteps across all types rather than examining how these factors may impact the occurrence of specific misstep types. At this point, we do not yet know if the distinction between misstep types is important; thus, future research should explore the impact of different missteps on client engagement. Subsequent research might also pursue a granular exploration of how treatment planning, preparation, and session participant type may differentially impact the frequency of specific misstep types (e.g., *demanding attention*) over others (e.g., *advice-giving*). In addition, given that therapists who are perceived as culturally understanding were found to significantly predict multiple dimensions of treatment engagement regardless of youth race and racial matching (Chu et al., 2022), future studies could examine how specific misstep occurrences that may influence perceived levels of cultural understanding and humility (e.g., giving *advice* that is inconsistent with cultural values of the youth or caregiver) could differentially impact dimensions of treatment engagement.

Similarly, while the present study identified several distal or proximal risk factors that predicted a higher occurrence of missteps, associations for other proximal factors, such as the preceding therapist’s use of specific structured clinical procedures, were not examined. Although the findings provide a descriptive picture of when certain misstep types were found to occur within a treatment event (e.g., *elicit perspectives about the problem* often preceded misstep occurrences), the present study did not examine whether specific clinical procedures increased the likelihood of misstep occurrences (e.g., examining whether *elicit perspectives about the problem* confers higher risk for a misstep occurrence). Further research examining associations between the specific structured clinical procedures identified in the present study and occurrences of specific misstep types may clarify best practices for improving training and supervision.

Most notably, one primary limitation of the present study is that it did not directly examine the impact of these potential therapist misstep occurrences on relevant outcomes, including how these missteps may negatively and differentially impact youth and family engagement within- and across-treatment events (e.g., immediate youth and caregiver reactions to session missteps, session attendance, changes in reported treatment engagement on a survey), as well as how they may disrupt therapist delivery of care (e.g., extensiveness of evidence-based practice delivery) were also not examined. These analyses are crucial for understanding whether and how therapist missteps may impact domains of treatment engagement and treatment delivery. However, the study’s novel approach of first populating a set of potential therapist missteps and methodically examining the nuances of their occurrence before examining their impact on relevant outcomes poses many strengths. In contrast to previous research, which often utilizes poor outcomes (e.g., client indicators of alliance ruptures, premature termination) to identify precipitating factors (e.g., therapist contribution to poorer engagement), the present study poses a unique contribution by anchoring to specific therapist behaviors, regardless of how youth, caregivers, and therapists subsequently respond. This approach addresses several crucial gaps in the current literature, including a paucity of focus on therapist behaviors that is inconsistent with the dyadic nature of engagement (Eubanks et al., 2019), concerns of under-detection engagement challenges by relying solely on client behaviors that may be minimized or concealed (Eubanks et al., 2018; Safran & Kraus, 2014), and prior findings suggesting that multifinality with regards to impact of missteps is highly likely (Park et al., 2024) given that clients may experience a range of responses (or a lack thereof) to occurrences of missteps across specific types. Building on the present findings, future analyses are underway to examine the potential impact of therapist missteps on youth and caregiver treatment engagement and the quality of evidence-based treatment delivery to produce actionable findings to improve therapist training and supervision in community mental health settings.

## Conclusion

Evidence has long documented the impact of therapist behaviors on client outcomes, with researchers highlighting the importance of identifying what other relevant dimensions of therapist behaviors may occur throughout treatment delivery that have gone unmeasured and are yet to be discovered (King & Bickman, 2017). The present study makes a unique contribution to the field aimed at understanding the potential for inadvertent harm in current youth and family mental health services by identifying distal and proximal risk factors associated with more frequent occurrences of missteps. Although the impact of these missteps on youth and family engagement was not explored in the present study, growing research in this area has outlined the potential negative impact of therapist missteps on youth and caregiver treatment engagement and treatment delivery, which has implications for treatment gains and outcomes for families seeking services. Building on this work, future studies should examine the association between therapist missteps and key treatment outcomes, including youth and caregiver engagement within- and across sessions. Overall, these findings have important implications for clinical practice and supervision in community mental health settings. Allocating clinical supports and organizational resources to enhance therapist preparedness, reducing days elapsed between supervision and treatment, and addressing the unique dynamics of family therapy sessions may reduce the occurrence of missteps and improve the quality of community mental health services for youth and families.
